# Reverse Immunology Approach to Define a New HIV-gp41-Neutralizing Epitope

**DOI:** 10.1155/2019/9804584

**Published:** 2019-03-24

**Authors:** Karim Dorgham, Nicolas Pietrancosta, Amel Affoune, Olivier Lucar, Tahar Bouceba, Solenne Chardonnet, Cedric Pionneau, Christophe Piesse, Delphine Sterlin, Pablo Guardado-Calvo, Philippe Karoyan, Patrice Debré, Guy Gorochov, Vincent Vieillard

**Affiliations:** ^1^Sorbonne Université, INSERM, CNRS, Centre d'Immunologie et des Maladies Infectieuses-Paris (CIMI-Paris), F-75013 Paris, France; ^2^Université Sorbonne Paris Cité, CNRS, Laboratoire de Chimie et Biochimie Pharmacologiques et Toxicologiques, F-75006 Paris, France; ^3^Sorbonne Université, CNRS, Institut de Biologie Paris-Seine (IBPS), Service de Synthèse Peptidique et d'Interactions Moléculaires, F-75005 Paris, France; ^4^Sorbonne Université, INSERM, Plateforme Post-génomique de la Pitié Salpêtrière (P3S), F-75013 Paris, France; ^5^Assistance Publique-Hôpitaux de Paris (AP-HP), Groupement Hospitalier Pitié Salpêtrière, Département d'Immunologie, F-75013 Paris, France; ^6^Institut Pasteur, Unité de Virologie Structurale (VIST), F-75015 Paris, France; ^7^Sorbonne Université, Ecole Normale Supérieure, PSL University, CNRS, Laboratoire des Biomolécules, LBM, F-75005 Paris, France

## Abstract

The design of immunogens susceptible to elicit potent and broadly neutralizing antibodies against the human immunodeficiency virus type 1 (HIV-1) remains a veritable challenge in the course of vaccine development. Viral envelope proteins adopt different conformational states during the entry process, allowing the presentation of transient neutralizing epitopes. We focused on the highly conserved 3S motif of gp41, which is exposed to the surface envelope in its trimeric prefusion state. Vaccination with a W614A-modified 3S peptide induces in animals neutralizing anti-HIV-1 antibodies among which we selected clone F8. We used F8 as bait to select for W614A-3S phage-peptide mimics. Binding and molecular docking studies revealed that F8 interacts similarly with W614A-3S and a Mim_F8-1 mimotope, despite their lack of sequence homology, suggesting structural mimicry. Finally, vaccination of mice with the purified Mim_F8-1 phage elicited HIV-1-neutralizing antibodies that bound to the cognate W614A-3S motif. Collectively, our findings provide new insights into the molecular design of immunogens to elicit antibodies with neutralizing properties.

## 1. Introduction

Despite the success of antiretroviral therapy, which has turned human immunodeficiency virus type 1 (HIV-1) infection into a chronic disease and has reduced the number of new infections worldwide, a vaccine against HIV-1 is still urgently needed. Due to its high mutability (1–10 mutations/genome/replication cycle), HIV-1 has evolved a unique arsenal of tricks to evade the immune system. Other mechanisms of immune evasion by HIV-1 are mediated by the nature of the native envelope (Env) spikes on the viral surface that mediate infection through receptor binding and fusion and that are the major targets for virus-neutralizing antibodies (Abs) [1]. Finally, the trimeric Env spike does not have a fixed conformation but is characterized by a tendency to breathe, resulting in tremendous flexibility with a native closed form, shifting toward more open conformations. In agreement with this model, while breathing, some neutralizing epitopes that are not accessible in the native state become available in the relaxed conformation (e.g., the CD4 binding site epitope recognized by the IgG1b12), whereas other broadly neutralizing Abs, such as VRC01, lock down the spike in its native closed conformation suppressing further breathing [2].

Consistent with the difficulties encountered in attempts to induce broadly neutralizing Abs against HIV-1 in vaccination studies, most patients generate some level of neutralizing Abs, and after years of infection, these can mature into HIV-1 broadly neutralizing Abs in 10 to 25% of HIV-infected patients. These Abs are able to target more conserved regions of the Env and thus neutralize about 90% of circulating HIV-1 strains [3–9]. Several animal and human studies have highlighted their putative role in protective immunity after passive transfer, in which a major but transient effect on the viral load was observed, due to the rapid implementation of viral escape mechanisms [10–18].

Neutralizing Abs map to four major Env antigenic sites: the CD4 binding site, the V1-V2 glycopeptidic loop, glycan V3 structures, and some gp41 motifs. Although gp41 is more conserved than the gp120 subunit, neutralizing Abs targeting gp41 are rarely detected in patients [19]. Only peculiar conformations of gp41 might be prone to induce broadly neutralizing Abs [20–25]. In addition to the highly conserved gp41 membrane-proximal external region (MPER) [26] and the gp120/gp41 interface [27], a conserved and protective motif, called 3S, localized between the N-terminal heptad repeats (HR) 1 and HR2 has been described [28, 29]. Vaccination of SHIV-challenged macaques with the 3S motif leads to immune protection and restores immune homeostasis, although anti-3S Abs do not neutralize the virus [30, 31]. An alanine-scanning assay identified the W614 position into the 3S motif as crucial for the virus entry although the W614A-3S mutant peptide is nevertheless able to elicit cross-clade-neutralizing Abs (nAbs) in vaccinated mice [32] as well as in rabbit and macaque models (Vieillard et al., unpublished data). Accordingly, amino-acid changes into gp41 MPER induce viral neutralization sensitivity [33]. Interestingly, among HIV-1-infected patients, natural anti-W614A-3S Abs were detected in less than 5% of progressors [32], but up to 25% of long-term nonprogressors (LTNP) [34]. The neutralizing capacity of W614A-3S Abs was inversely correlated with viral load and viral DNA and was associated with the preservation of high CD4+ T-cell counts and T-cell responses in LTNP patients [34].

We postulated that W614A-3S-specific nAbs could play a role in the maintenance of the non-progressor status and that they could be used to select for anti-HIV immunogens with improved activity. Analysis of HIV-1-Env trimer structures [35, 36] highlighted the 3S area being part of a flexible loop, which ensures the junction between strand *β*27 (Leu602_gp41_-Thr606_gp41_) and helix *α*8 (Leu619_gp41_-Trp623_gp41_) in the gp41-prefusion structure and between HR1 and HR2 helices in the postfusion state of gp41. Rearrangements between each conformational state require C*α*-movements from either side of the linker in which the 3S motif is anchored. No structural data are presently available regarding the conformation of the neutralizing epitope recognized by anti-W614A-3S Abs, and we can only infer that this epitope could also be conformation dependent. The goal of this study was therefore to better characterize the molecular capability of the W614A-3S epitope using molecular modeling and reverse immunology approaches.

**Figure 1:**
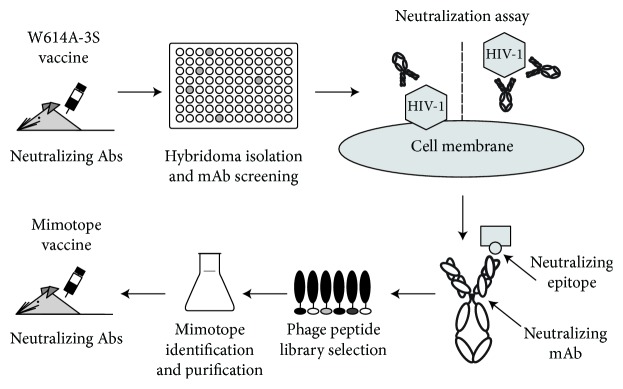
Reverse immunology applied to an epitope-based vaccination strategy. Mice immunized with the W614A-3S peptide were used to generate mAbs. Clones that reacted with the vaccine peptide were studied for their HIV-1-neutralizing properties. The neutralizing F8 mAb was then used to screen a phage peptide library for mimotopes of the F8 epitope. Finally, vaccination of mice with the selected mimotope induced neutralizing Abs in the serum.

In this work, a murine anti-W614A-3S mAb with HIV-1-neutralizing properties was isolated and then used to screen a phage peptide library for W614A-3S mimotopes. Phage-mimotope vaccination led to the generation of antibodies that bind the cognate W614A-3S vaccine associated with neutralizing activity in mice (Figure 1).

## 2. Materials and Methods

### 2.1. Mouse Vaccination

All experiments were undertaken by experienced and authorized staff, following health and safety procedures established according to French legislation governing the use of animals in experiments. Mice were immunized with the W614A-3S peptide conjugated to KLH (KLH-W614A-3S) with MBS (m-maleimidobenzoyl-*N*-hydroxysuccinimidyl ester), and serum IgG titers were evaluated by ELISA, as previously reported [32]. Fusion and production of mAbs were performed by the HT-MAb platform (Sysdiag-UMR3145, Montpellier, France). Briefly, mice received a final i.v. boost injection two weeks after the previous immunization. Four days later, the animals were euthanized and B cells obtained from the spleen were fused with the SP2/0 cell line (ratio 5 : 1) using polyethylene glycol. Hybridoma cells were selected in HAT media supplement (Sigma), and culture supernatants were screened by indirect ELISA with BSA-W614A-3S as coating molecule. Finally, selected hybridomas were subcloned five times to obtain stable Ig-producing cells and maintained in culture in DMEM enriched with L-glutamine, pyruvate, and 4.5 g/L glucose, supplemented with 100 *μ*g/mL gentamicin and with 10% inactivated fetal bovine serum. IgGs were purified from culture supernatant by protein G chromatography.

Immunization of mice with the Mim_F8-1 phagotope was realized by CovalAb (Villeurbanne, France). Six- to eight-week-old BalB/c mice were immunized four times at two weekly intervals subcutaneously with 10^10^ cfu of the inactivated Mim_F8-1 phagotope according to the protocol described in Supplemental Figure 1.

### 2.2. Enzyme-Linked Immunosorbent Assay (ELISA)

Ninety-six-well Maxisorp immunoplates (NUNC) were coated overnight at 4°C with 0.2 *μ*g/well of peptide conjugated with the carrier protein in PBS. After three washes in PBS, plates were incubated for 1 hour at 37°C with 4% PBS low-fat milk (PBSM). Dilutions of mAbs or serum from immunized mice were blocked with 1 vol. of PBSM and incubated in plates for 1 hour at 37°C. Plates were then washed three times with PBS 0.05% Tween 20 (PBST) followed by three more washings with PBS and wells incubated for 1 hour at 37°C with 100 *μ*L of HRP-conjugated anti-mouse IgG (Sigma, #A9917).

### 2.3. Neutralization Assays

Neutralization of the purified Abs was tested at various concentrations, as described [32, 34] using the TZM-bl reporter cell line. At 48 hours after infection, luciferase activity in TZM-bl lysates was measured using the Bright-Glo™ Luciferase Assay Substrate (Promega). A cutoff value of 2.5 times background was applied to determine positive values. Total IgG from mouse sera were purified with the Melon Gel IgG Purification System (Thermo Scientific), and concentrations were determined by ELISA. Depletion of anti-W614A-3S Abs was done by incubating purified IgG with immobilized CRM-W614A-3S (10 *μ*g/mL) for 2 hours. The IC_50_ values were calculated by nonlinear regression analysis using Prism 6 (GraphPad Software).

### 2.4. Biopanning of the Phage-Peptide Library

The phage-peptide library [37] was kindly provided by Igor Fisch and Greg Winter (MRC, Centre for Protein Engineering, Cambridge, UK). The phage library was selected using 50 *μ*g of mAb, as previously reported [38]. After the third round of biopanning, isolated clones were picked randomly from LB agar plates and used in PCR for DNA insert amplification and sequencing. In parallel, colonies were grown overnight at 30°C in 200 *μ*L of 2YT/tetracycline and the supernatant used directly for phage ELISA.

The selected F8 phagotope was PEG purified from 1 L culture supernatant, resuspended into 10 mL water, and filtered through a 0.45 *μ*m filter. The suspension was then buffer exchanged with PBS by ultrafiltration using Amicon Ultra 2 mL centrifugal filters MWCO 100 kDa and concentrated 10 times. Phages were heat inactivated at 72°C for 4 hours and frozen in aliquot to be used subsequently for mice immunization.

### 2.5. Molecular Modeling

The crystal structure of the HIV-1 BG505 SOSIP.664 Env trimer ectodomain, comprising atomic-level definition of prefusion gp120 and gp41, in complex with human antibodies PGT122 and 35O22 was used as the template (pdb code 4TVP) [35]. The homology model of antibodies was built using the model full-length antibody protocol of discovery studio 4.5. The protocol requires the sequences for the light and heavy chains of the antibody to be modeled, which are duplicated to generate the sequence for the full-length antibody. The Immunoglobulin G based on templates for IgG (pdb code: 1igy) was used for the homology model [39]. The alignment was then used to build homology models using Modeler. The best model of the 50 generated was selected based on its Modeler PDF energy and DOPE Scores.

The 3D structures of mimotopes were designed using the de novo peptide structure prediction server PEP-FOLD [40]. The five most probable 3D structures were retained for docking experiments. Mimotopes were docked into the homology model using the ZDOCK protocol using discovery studio interface. ZDOCK is a rigid-body protein-protein docking algorithm based on the fast Fourier transform correlation technique that is used to explore the rotational and translational space of a protein-protein system. The poses were refined with RDOCK, a CHARMm-based energy minimization procedure for refining and scoring docked poses using energy scoring functions [41]. The best poses were selected to analyze the binding pattern of peptides.

## 3. Results

### 3.1. Molecular and Functional Characterization of Anti-W614A-3S mAbs

Serum from mice immunized with KLH-W614A-3S peptide showed a significant neutralizing activity on cross-clade HIV-1 strains, as reported in reference [32]. Monoclonal antibodies (mAbs) isolated from these mice were studied in this work. Two fusion experiments were realized, and 1152 supernatants of hybridoma were screened by indirect enzyme-linked immunosorbent assay (ELISA). Twelve wells showed a specific binding on the W614A-3S conjugated to BSA. After stabilization by repeated cell cloning, five hybridomas (B8, C9, F8, G6, and G9) were analyzed to characterize the secreted mAbs at the molecular level. All mAbs were of the IgG1 isotype, and the sequence analysis of their variable domains revealed that the F8 and C9 clones secreted the same mAb (hereafter called F8), whereas B8 and G9 clones secreted identical mAbs (hereafter called B8), leading to the identification of three different mAbs (B8, F8, and G6; Supplemental Table I). It is of note that the B8 mAb shares the same variable light chain with the G6 mAb, using the IGKV1-117 and IGKJ5 genes to form the junction, while the Vk domain of the F8 mAb used GKV1-117 and IGKJ2 genes. Mass spectrometry analysis of the F8 mAb validated the sequence of the light chain (Supplemental Figure 2). Furthermore, the variable domains of the heavy chains of the three mAbs were very different in terms of the V/D/J gene family used in the junction and regarding the amino acid sequences of the CDR1, 2, and 3 (Supplemental Table I). The F8 CDR3 loop is consisting of only five residues (YGYGY), according to the IMGT unique numbering for V domain [42].

**Figure 2:**
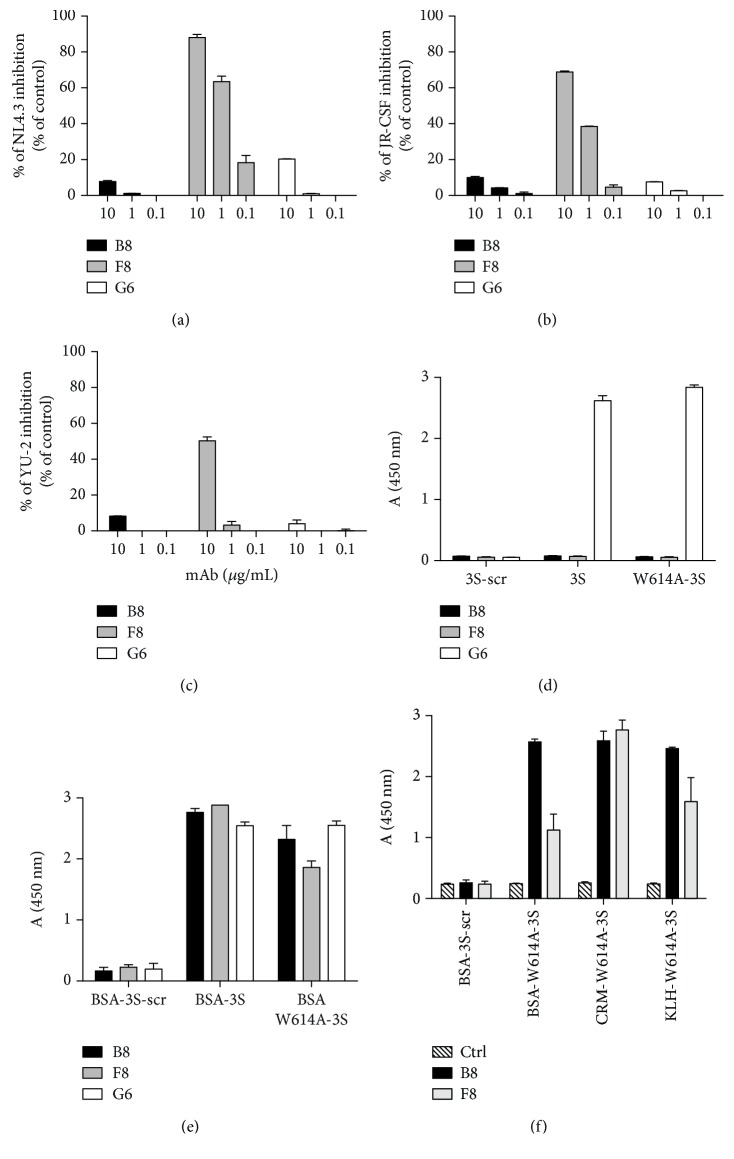
Functional characterization of the anti-W614A mAbs. Neutralization of HIV infection with anti-W614A-3S mAbs is shown in (a–c). Dose response of neutralizing activity was performed with a standard TZM-bl assay against tier 1 (NL4.3) and tier 2 (JR-CSF, YU-2) HIV-1 clade B strains, with the purified anti-W614A-3S mAbs. Data are presented as percent infection compared to the maximum signal (100%) into the positive control (infected cells in medium without mAb). Experiments were run in duplicates and bars indicate SD. Characterization of the binding properties of the anti-W614A-3S mAbs is shown in (d–f). Purified anti-W614A-3S mAbs were tested by ELISA at 1 *μ*g/mL on immobilized free peptides (d) and immobilized BSA-conjugated peptides (e). The 3S-scrambled peptide (3S-scr) functioned as negative control for those experiments. (f) illustrates reactivity of the B8 and F8 mAbs in ELISA with immobilized W614A-3S conjugated to different carrier proteins (BSA: bovine serum albumin, CRM: CRM197, and KLH: keyhole limpet hemocyanin). The mouse IgG1 isotype mAb (Ctrl) functioned as negative control (hashed bars). Binding activity of mAbs is expressed as mean OD at 450 nm of duplicate wells, and bars indicate SD. These data are representative of three independent experiments. B8 activity and F8 activity are indicated as black and gray columns, respectively, in all panels. G6 activity is indicated as white columns in (a–e).

The three mAbs (B8, F8, and G6) were then tested for their HIV-1-neutralizing potential against tier 1 (NL4.3) and tier 2 (JR-CSF and YU-2) HIV-1 clade B strains (Figures 2(a)–2(c)). F8 only could neutralize NL4.3, JR-CSF, and YU-2 strains, with IC_50_ values of 0.4, 2.4, and 9.7 *μ*g/mL, respectively. F8 was also capable to neutralize ADA, TRO.11, QHO692.44 HIV-1 clade B, and ZM249M.Pl1 HIV-1 clade C strains, with IC_50_ values of 1.7, 2.3, 3.1, and 4.7 *μ*g/mL, respectively (Table 1). As a control, a chimeric Ab, connecting the variable murine domains of the F8 mAb with the constant domains of a human IgG1, was constructed (Supplemental methods and supplemental Figure 3.A). The F8 chimeric Ab preserved the neutralizing activity of the parent murine mAb (Supplemental Figure 3.B), thus demonstrating that the identified variable domains mediate neutralization, regardless of the context provided by constant Ig domains.

The different neutralizing properties of the mAbs suggest that they recognize different epitopes presented by the immunogenic peptide used for immunization. Thus, their binding capacities were further studied by ELISA. The G6 mAb interacts with both the unconjugated wild-type 3S and W614A-3S peptides, but not with the randomly scrambled control sequence peptide (3S-scr), while neither F8 nor B8 mAbs bind these three peptides (Figure 2(d)). As expected, all mAbs bind to the BSA-coupled W614A-3S peptide but not the control BSA-3S-scr peptide. It is of note that they also interact with the BSA-3S peptide (Figure 2(e)). These results indicate that G6 mAb binds a linear epitope, whereas B8 and F8 mAbs both recognize a conformational epitope. To investigate the contribution of the carrier protein in the peptide presentation, BSA was exchanged with KLH or CRM197, a detoxified form of diphtheria toxin. Both the B8 and F8 mAbs recognize W614A-3S peptide independently of the carrier protein, as shown in Figure 2(f). Interestingly, none of the isolated mAbs interacted with soluble recombinant gp160MN/LAI-2 [43] in ELISA (data not shown), suggesting that neither the linear nor the conformational epitopes, recognized by the W614A-3S mAbs, are accessible into the monomeric gp160.

Whereas immunization of mice with the wild-type 3S peptide of gp41 did not induce neutralizing Abs [30, 31], generation of the F8 mAb after vaccination with the W614A-3S peptide suggests that the neutralizing epitope is associated with the W614A-3S point mutation introduced in the 3S motif. Comparative kinetics and apparent affinity measurements of F8 mAb binding to 3S or W614A-3S were performed by surface plasmon resonance (SPR). While apparent rates of association were similar, the calculated affinity constant (KD) was higher for BSA-3S than for BSA-W614A-3S because of a more rapid F8 mAb dissociation rate from the former peptide (Supplemental Figure 4).

### 3.2. Characterization of the Neutralizing Epitope by Reverse Immunology

**Figure 3:**
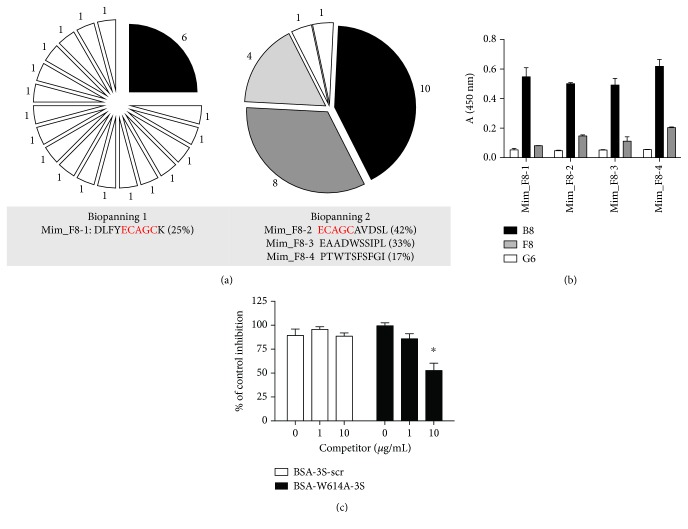
Phage-mimotope selection and reactivity of the anti-W614A-3S mAbs. A phage library of decapeptides was screened with the F8 mAb (a). After three rounds of selection in the first biopanning, the mim_F8-1 phagotope represented 25% of the selected clones. In a second experiment of biopanning, three phagotopes were identified at the third round of selection. Mim_F8-2 represented 42% of the selected clones and shared a five-residue motif with those of Mim_F8-1. (b) Binding properties of the selected phagotopes were tested by ELISA on immobilized anti-W614A-3S mAbs. Binding activity is expressed as mean OD at 450 nm of duplicate wells and bars indicate SD. These data are representative of three independent experiments. (c) Competitive binding ELISA of Mim_F8-1 on the immobilized F8 mAb with indicated concentrations of BSA-W614A-3S (black bars). BSA-3S-scr functioned as negative control (white bars). Results are expressed as percentage of binding of the Mim_F8-1 without competitor (100%). Values are the means of two different experiments with two replicated measurements, and bars indicate SEM. ^∗^ *p* < 0.05, according to Mann–Whitney *U* test.

Phage display technology has proven to be an effective and practical technique for the identification of linear or conformational epitopes and has been widely used for mapping HIV-1 epitopes [44]. To characterize the neutralizing conformational epitope of the immunogenic W614A-3S peptide, a phage peptide library was screened with the F8 mAb. After four rounds of biopanning, DNA sequencing of the selected clones led to the identification of a single sequence (denoted Mim_F8-1). This major clone was also found in the third round of selection with 25% enrichment (6 of 24 clones). A new biopanning experiment was performed, using less drastic washing conditions, to select other mimotopes. Finally, after three rounds of selection, three supplemental mimotopes could be isolated and a preferentially selected clone (denoted Mim_F8-2) was found to have a 42% enrichment level (10 of 24 clones). It is noteworthy that the clone Mim_F8-1 was not recovered in the second biopanning experiment; however, its sequence shared a common five-residue motif “ECAGC” with that of Mim_F8-2 (Figure 3(a)), suggesting identification of the F8 mAb epitope, regardless of sequence homology with BSA-W614A-3S (supplemental Table II).  Figure 3(b) shows that all selected mimotopes bound the F8 mAb, but not the non-neutralizing G6 and B8 mAbs. To evaluate the capacity to compete with W614A-3S for binding to the F8 mAb, the Mim_F8-1 phagotope (i.e., phage-bearing mimotope) was incubated with plate-immobilized Abs in the presence of various concentrations of the BSA-coupled W614A-3S peptide and phage binding was measured by ELISA.  Figure 3(c) shows that the phagotope interaction with F8 is inhibited in a dose-dependent manner with BSA-W614A-3S but not with BSA-3S-scr (used as negative control), suggesting competition between the phagotope and BSA-W614A-3S for binding to the F8 mAb. The F8 mAb did not interact with the synthetic-free peptides corresponding to the sequence mimotopes, neither in direct ELISA nor in competition assay with the phagotopes (data not shown). Altogether, these results could suggest that peptide mimotopes adopt a constrained conformation at the phage surface, allowing binding to the F8 mAb.

In the context of the phage, the C-terminal extremity of the mimotope is fused to the pIII coat protein, leaving its N-terminal extremity free to move. A Mim_F8-1 peptide was therefore synthesized using a biotinylated resin to produce a peptide biotinylated exclusively at the carboxy terminus in an attempt to mimic peptide phage display. Furthermore, a disulfide bridge was created by oxidation of the two Mim_F8-1 cysteines, to investigate the role of a potential loop between these amino acids. A study of interactions by biolayer interferometry (BLI), using a streptavidin biosensor to immobilize the biotinylated Mim_F8-1 peptide, showed that the F8 mAb was unable to bind either the linear or the cyclic Mim_F8-1 peptides, as compared to a control mAb (Supplemental methods and data not shown). Finally, to investigate the impact of multivalent presentation of the mimotope for the binding to the F8 mAb, complexes between biotinylated Mim_F8-1 peptide and streptavidin beads were constructed. Monitoring of interactions by BLI using an anti-mouse IgG Fc capture biosensor to immobilize the F8 mAb did not allow detection of specific binding with those complexes of linear or cyclic Mim_F8-1 peptides (data not shown). These results suggest that only pIII-fused mimotopes will be able to bind the F8 mAb and to induce the cognate antibody *in vivo*.

### 3.3. Validation of the Neutralizing Epitope by Mice Immunization

**Figure 4:**
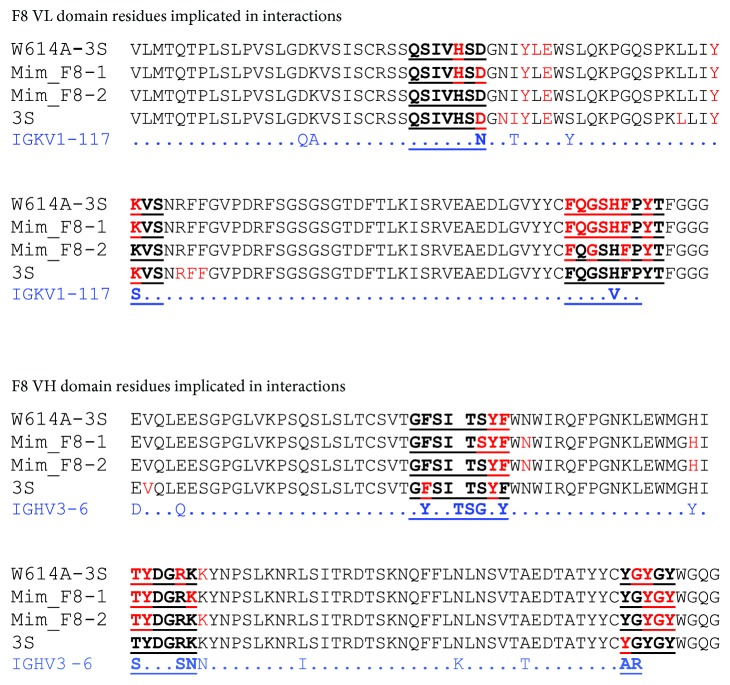
Molecular interaction between the F8 mAb and peptides. The target peptides are indicated in the first column. The amino acid sequences of both the VL and the VH domains are indicated in the second column. CDRs are underlined and indicated in bold letters while residues involved in the interaction with the peptide are indicated in red. The closest F8 germline-encoded V regions, identified in Supplemental Table I, are indicated in blue.

**Figure 5:**
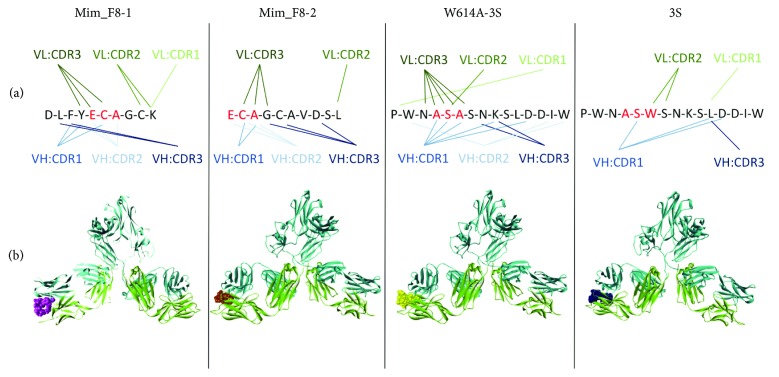
*In silico* docking studies of the 3S derivatives with F8 mAb. Predominant molecular interactions between peptide residues and F8 CDRs are indicated in (a) for each antigen, and the binding interactions of the peptides (hard spheres) with the F8 mAb (cartoon representation) are illustrated in a 3D model (b).

Regarding the F8 mAb, 24 residues are mutated from the closest germline-encoded V regions, including 3 and 10 amino acids into the CDRs of the VL and VH domains, respectively (Figure 4). The molecular docking approach was used to compare the structural interactions of the different peptides with the F8 mAb. The data indicate that 13 and 8 residues from the VL and VH domains, respectively, interact with W614A-3S. The Mim_F8-1 mimotope, as well as the W614A-3S peptide, interacts with all CDRs of both the VH and VL domains (Figure 5). As shown in Figure 4, the predicted interactions of these peptides with the F8 mAb are very similar and involve six residues closely juxtaposed in the light chain CDR3 (CDR L3) and three and two residues of the CDR H3 for Mim_F8-1 and W614A-3S, respectively. From the opposite point of view, F8 CDR residues are predicted to interact mainly around the alanine residue, corresponding to the W614A mutation into the 3S derivative peptide and around the ECAGC motif of Mim_F8-1, as shown in Figure 5. The predicted interactions involved in antigen binding of Mim_F8-2 with F8 are slightly different, with a non-substantial reduced number of interactions (Figures 4 and 5). In conclusion, the three peptides are predicted to be anchored in the binding site of the F8 mAb as illustrated by the 3D model shown in Figure 5(b).

It is of note that the docking prediction of the 3S peptide, based on the BG505 SOSIP structure, is very different from that of the W614A-3S peptide, with the engagement of only few residues of four CDRs of the F8 mAb (Figures 4 and 5). Overall, *in silico* analysis suggests that the W614A mutation in the 3S motif generates a conformational change, giving its shape to the epitope and allowing a better interaction with F8 mAb.

**Figure 6:**
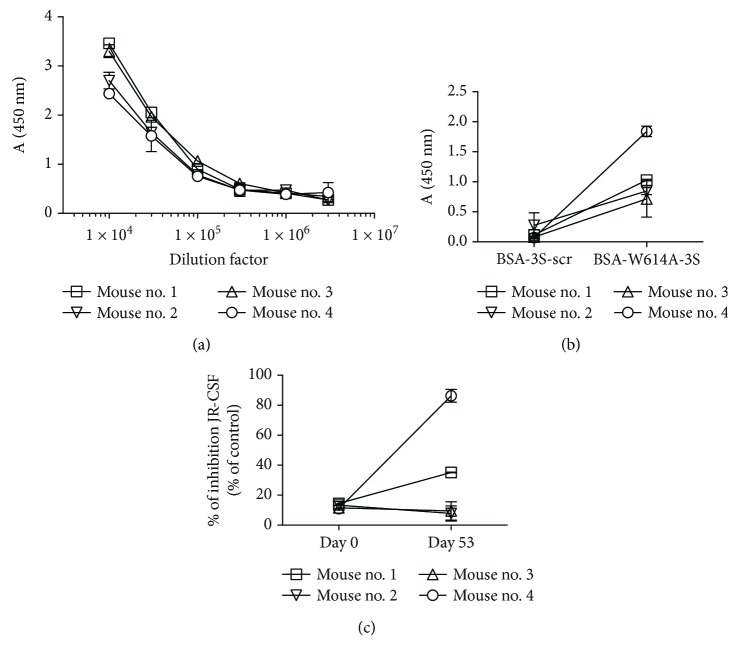
Serum reactivity of mice immunized with the Mim_F8-1 phagotope. (a) Titers of serum IgG directed at phage proteins were determined by ELISA against immobilized Mim-F8 phagotope. (b) IgG binding reactivity of the sera (1 : 50 dilution) against immobilized BSA-W614A-3S was assayed by ELISA at day 53. BSA-3S-scr functioned as negative control. Binding activity is expressed as mean OD at 450 nm of duplicate wells and bars indicate SD. (c) Neutralizing activity of the sera (1 : 2 dilution) at day 53 was performed as mentioned in Figure 1 using the JR-CSF strain. Sera at day 0 were used as negative control. Experiments were run in duplicate and bars indicate SD.

**Figure 7:**
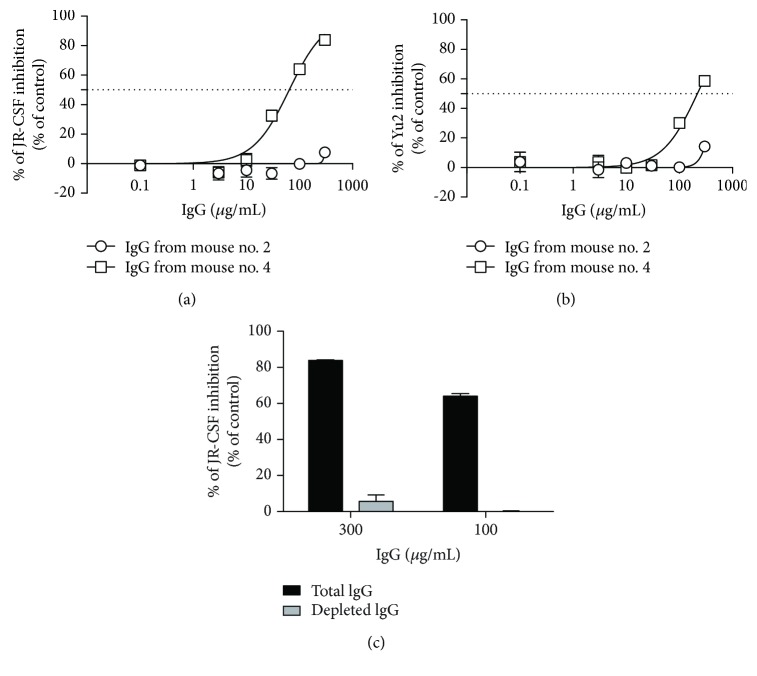
Neutralizing properties of purified IgG from mice immunized with the Mim_F8-1 phagotope. Dose-response curves of total purified IgG from poor (Mouse no. 2) and good (Mouse no. 4) responder mice tested to neutralize JRCSF (a) and YU-2 (b) HIV-1 strains. Neutralizing activity was performed as in Figure 1. Experiments were run in triplicate and bars indicate SD. (c) Reactivity of the purified IgG at 100 and 300 *μ*g/mL from the good responder mouse, before (black bar) and after depletion of the W614A-3S Abs (grey bar). Neutralizing activity was performed as above using the JR-CSF strain. Experiments were run in duplicate and bars indicate SD.

Finally, to obtain proof of concept of its immunogenic potential, four mice were immunized with Mim_F8-1 phagotope (Supplemental Figure 1). As expected, all showed robust amounts of anti-phage Abs at day 53, with end-point titers reaching a 10^5^ dilution factor (Figure 6(a)). Interestingly, anti-W614A-3S IgG were also detected by ELISA at days 39 and 53, at titers that varied among the immunized mice (Figure 6(b)). More importantly, among the four immunized mice, the one with the most anti-W614A-3S Abs displayed neutralizing activity against the tier 2 JR-CSF HIV-1 strain with up to 80% of inhibition (Figure 6(c)). Purified IgG from a poor responder mouse did not neutralize the virus (IC_50_ > 300 *μ*g/mL for JRCSF and YU-2), while purified IgG from a good responder mouse had IC_50_ values of 49 and 218 *μ*g/mL for JRCSF and YU-2, respectively (Figures 7(a) and 7(b)). To confirm the involvement of the anti-W614A-3S Abs in the neutralization process, purified IgG were adsorbed on CRM-W614A-3S. Anti-W614A-3S depleted IgG from the good responder mouse failed to neutralize the JRCSF strain (IC_50_ > 300 *μ*g/mL), as shown in Figure 7(c).

## 4. Discussion

The identification of the molecular specificities of Abs that mediate neutralizing breadth and potency is key in the design and development of suitable Env-based immunogens for vaccination. The epitope-based vaccine design consists in grafting an epitope of interest onto a heterologous protein scaffold. Numerous epitope-scaffold designs, focusing MPER, CD4 binding, or glycan V3 sites of vulnerability, have been tested without eliciting cross-reactive serum neutralization of most primary strains of HIV-1 [45–47]. Recently, structure-based optimization of an epitope-based vaccine design provided fusion peptide-directed antibodies that neutralize diverse strains of HIV-1 [24]. In the present study, we sought to determine the molecular specificity of mAbs generated by immunization with the W614A-3S-modified peptide derived from gp41. Among the three mAbs tested for their neutralizing activity, two were non-neutralizing, as previously reported with the wild-type 3S peptide [30, 31], whereas the F8 mAb showed moderate neutralizing activity against tier 1 and tier 2 HIV-1 clade B and clade C strains with values of IC_50_ that are similar to those described for the human anti-gp41 MPER 2F5 and 4E10 mAbs [48, 49]. In SPR experiments, the F8 mAb exhibited similar association rates (kon) with BSA-coupled W614A-3S and wild-type 3S peptides but the dissociation rate (koff) of BSA-W614A-3S is significantly slower. Thus, apparent constant affinity (KD) of the F8 mAb is very low, reflecting its high affinity for this epitope. Similar observations have been described previously with various human and mouse mAbs, where koff values are highly predictive of anti-HIV-neutralizing activity [50]. This suggests that the F8 mAb recognizes different binding sites on wild-type 3S and W614A-3S peptides. This is highlighted in *in silico* molecular modeling studies of the F8 mAb that show different docking interactions with both peptides, illustrating the dramatic impact of the W614A mutation on the epitope conformation.

In the structure of the HIV-1 envelope trimer [35, 36], the 3S motif appears to be exposed at the surface in the trimeric prefusion structure of the HIV-1 envelope [34] and possibly adopts different conformations during the viral entry steps. This suggests that the neutralizing F8 mAb could recognize a conformational intermediate epitope [2, 51, 52] mimicked by the W614A-3S peptide. It has been reported that few neutralizing Abs targeting gp41 bind transient epitopes exposed during the conformational changes of the Env protein in the course of the viral entry process [24, 53, 54]. The W614 residue is highly conserved into the 3S motif of gp41 in all HIV-1 strains. Thus, the generation of W614A-3S Abs is very unlikely to result from the occurrence of dominant W614A-3S mutant viral strains in HIV-infected individuals, since they are non-infectious [32]. Anti-W614A-3S cross-reactive Abs would rather have been generated in the process of somatic hypermutation generated by non-W614A-3S-bearing strains.

To gain insight into the characterization of the W614A-3S nAbs, a phage peptide library [37] was screened with the neutralizing F8 mAb to select specific mimotopes. The preferentially selected mimotope (Mim_F8-1) interacts with F8, but not with the non-neutralizing mAbs, and competes with the W614A-3S peptide for the binding to the F8 mAb. These data suggest that W614A-3S and Mim_F8-1 share a similar conformation, even if there is no sequence homology which is consistent with the notion that most neutralizing Abs have conformational epitopes [55, 56]. Molecular docking indicated that the W614A-3S peptide and the selected mimotopes adopt the same conformation: they are anchored in the binding site of the F8 mAb, interacting with residues of all the CDRs of both the VH and the Vk domains. Notably, the CDR L3 of the F8 mAb is predicted to interact mainly with the mutated residue W614A into the 3S peptide and with the ECAGC motif of the mimotopes. It is now evident that the exposure of epitopes on HIV-1 and Env features increases nAb emergence; conversely, lack of neutralization is due to an inability of an antibody to access its epitope in the context of Env on the virus [57]. Consequently, structures capable of mimicking neutralizing transient epitopes, such as Mim_F8-1, might be useful in the molecular design of new immunogens. It is likely that interactions with the phage pIII protein constrain the peptide mimotope into a specific conformation, essential for binding to the F8 mAb [38, 58]. Therefore, the phage particle, or at least the pIII protein, should be considered an important element in vaccine formulation [59, 60]. Immunization of mice with the phage bearing the Mim_F8-1 mimotope led to the induction of anti-HIV-neutralizing Abs in the serum, demonstrating that structural mimicry of the Mim_F8-1 allows functional activity. It is of note that all immunized mice developed low titers of neutralizing Ab, which is certainly due to the strong response against the phage proteins. To increase the immunogenicity while retaining the constrained conformation imposed by the phage protein, the mimotope could be fused to soluble recombinant pIII and used as vaccine instead of a phagotope [61]. Finally, additional animal studies should be conducted, using rabbit and non-human primate models, to determine if immunization with Mim_F8-1 is superior to immunization with KLH-W614A-3S peptide in its capacity to elicit serum neutralization. At this point, our work is suggesting that it is indeed possible to preferably orientate an immune response towards the recognition of “F8-like” specificities, at the expense of “B8-like” or “G6-like” non-neutralizing responses.

Thus, in addition to revealing features of the W614A-3S motif that allows for neutralizing activity, the molecular survey of the F8 mAb provides new insight into molecular conformations of a highly conserved motif of gp41. Future structural studies will allow better understanding of the W614A-3S motif as a promising HIV-1 vaccine.

## Figures and Tables

**Table 1:** Neutralizing activity of F8 mAb on tier 1 and tier 2 HIV-1 clade B and clade C strains.

Virus	TZM-bl assay						
	NL4.3	JRCSF	YU-2	ADA	TRO.11	QHO692.44	ZM249M.Pl1
Tier	1	2	2	2	2	2	2
Clade	B	B	B	B	B	B	C
IC_50_ (*μ*g/mL)	0.4	2.4	9.7	1.7	2.3	3.1	4.7

## Data Availability

Sequences of F8 mAb reported in this paper have been deposited in the GenBank Database under accession numbers MK110657 and MK110658. All other data used to support the findings of this study are included within the supplementary information files or available from the corresponding author upon request.

## Supplementary Materials

Supplementary Materials Supplemental Figure 1: mice immunization protocol with the Mim_F8-1 phagotope. Supplemental Figure 2: sequence analysis of the F8 mAb by mass spectrometry. Supplemental Figure 3: construction and characterization of the chimeric F8 mAb. Supplemental Figure 4: SPR sensorgrams of F8 mAb binding to BSA-W614S-3S and BSA-3S. Supplemental Table I: molecular characterization of the anti-W614A-3S mAbs. Supplemental Table II: sequence of the peptides used in this study.
