# Supplementary material for: Reverse Immunology Approach to Define a New HIV-gp41-Neutralizing Epitope
Source: J Immunol Res. 2019 Mar 24;2019:9804584. doi: 10.1155/2019/9804584 (PMC6451809; doi:10.1155/2019/9804584)
Supplement: Supplementary Materials — Supplemental Figure 1: mice immunization protocol with the Mim_F8-1 phagotope. Supplemental Figure 2: sequence analysis of the F8 mAb by mass spectrometry. Supplemental Figure 3: construction and characterization of the chimeric F8 mAb. Supplemental Figure 4: SPR sensorgrams of F8 mAb binding to BSA-W614S-3S and BSA-3S. Supplemental Table I: molecular characterization of the anti-W614A-3S mAbs. Supplemental Table II: sequence of the peptides used in this study. [file 9804584.f1.pdf]

SUPPLEMENTARY INFORMATION

SUPPLEMENTAL FIGURES AND TABLES:

Supplemental Figure 1:

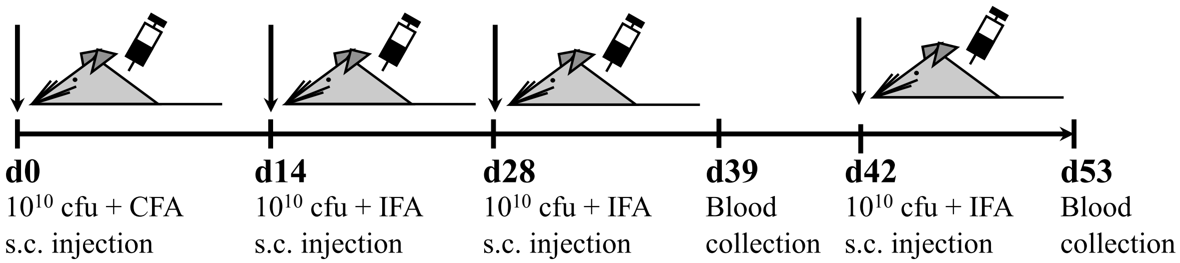

Supplemental Figure 1: Mice immunization protocol with Mim_F8-1 phagotope. Four BALB/c mice were injected subcutaneously with heat-inactivated Mim_F8-1 phagotope (10^{10} cfu) at day 0 in complete Freund's adjuvant (CFA) and at days 14, 28 and 42 in incomplete Freund's adjuvant (IFA). Serum was collected at days 39 and 53 and tested for their reactivity as described in Figure 5.

Supplemental Figure 2:

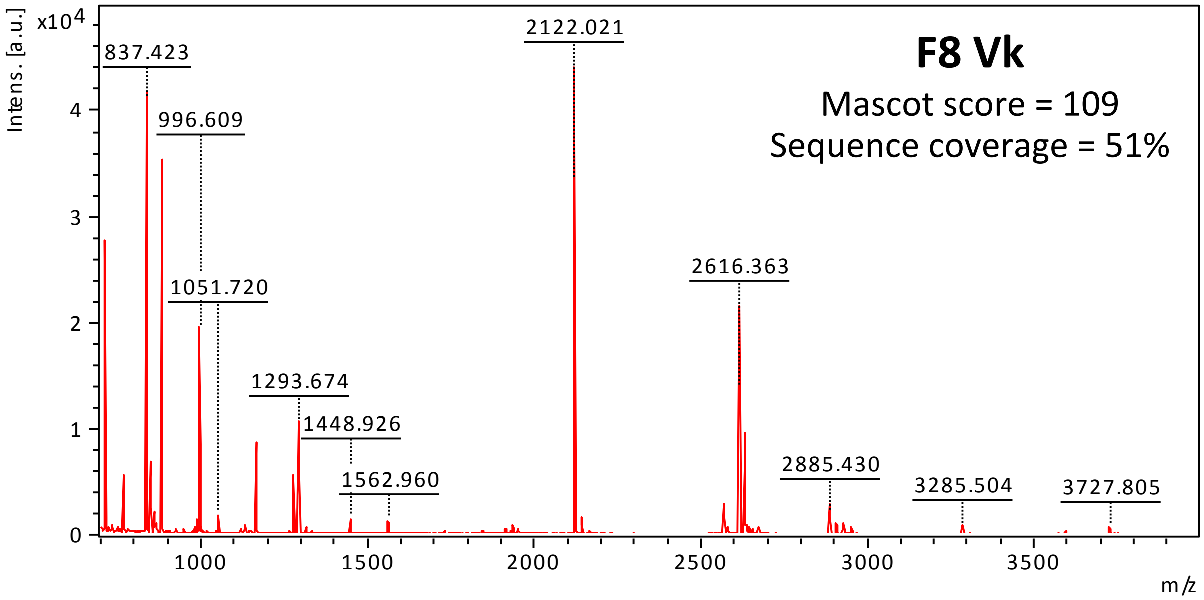

1 MKLPVRLLVL MFWIPASSSD VLMTQTPLSL PVSLGDKVSI SCR**SSQSIVH**

51 **SDGNIYLEWS** **LQKPGQSPK**L IY**KVSNRFFG** **VPDRFSGSGS** **GTDFTLKISR**

101 **VEAEDLGVYY** **CFQGSHFPYT** **FGGGTK**LELK **RADAAPTVSI** **FPPSSEQLTS**

151 **GGASVVCFLN** **NFYPK**DINVK **WKIDGSER**QN GVLNSWTDQD SKDSTYSMSS

201 TLTLTKDEYE RHNSYTCEAT HKTSTSPIVK SFNRNEC

Supplemental Figure 2: Sequence analysis of the F8 mAb by mass spectrometry. Identification of the light chain of the F8 antibody was made by MALDI-TOF PMF (peptide mass fingerprint). Spectra was obtained in positive reflector mode on an Autoflex speed IV (Bruker) in the mass range 700-4000 m/z using CHCA matrix. Identified peptides are shown in bold red in the sequence and CDRs of Vk are highlighted in yellow.

Supplemental Figure 3:

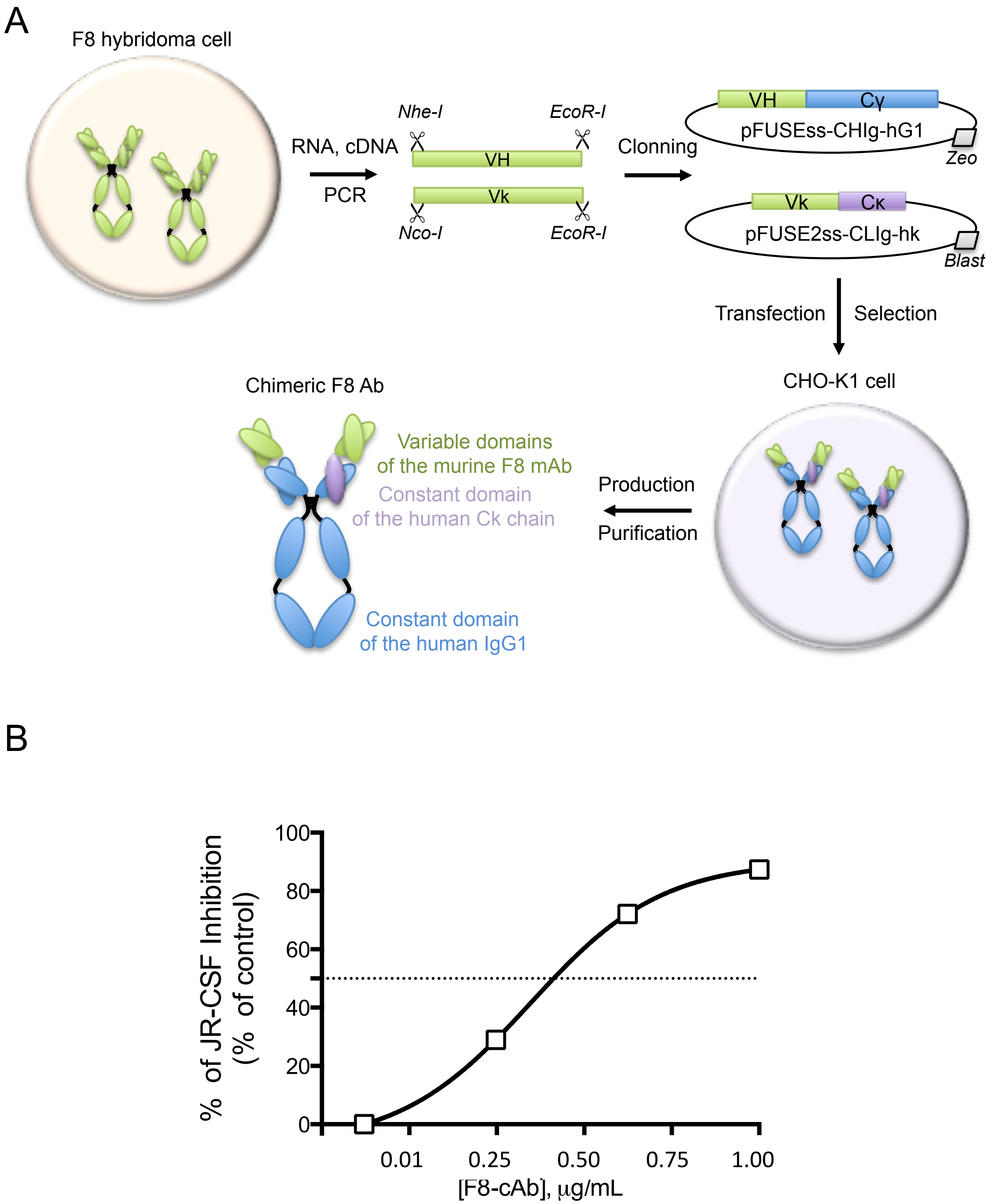

Supplemental Figure 3: Construction and characterization of the chimeric F8 mAb. (**A**) cDNA was synthesized after RNA extraction from F8 hybridoma. VH and VL domains were amplified by PCR with oligonucleotides coding for the indicated restriction sites. The DNA fragments were cloned into plasmids coding for the corresponding human constant IgG1 and k domains. CHO cells were co-transfected with the plasmids and selected with zeocin (zeo) and blasticidin (blast). The chimeric F8 mAb was purified using protein A agarose beads as mentioned in Supplemental information. (**B**) Neutralizing activity of HIV infection was performed as mentioned in Figure 2 using the JR-CSF strain and different concentrations of purified chimeric F8 mAb (F8-cAb). The calculated IC50 was 0.3 µg/mL.

Supplemental Figure 4:

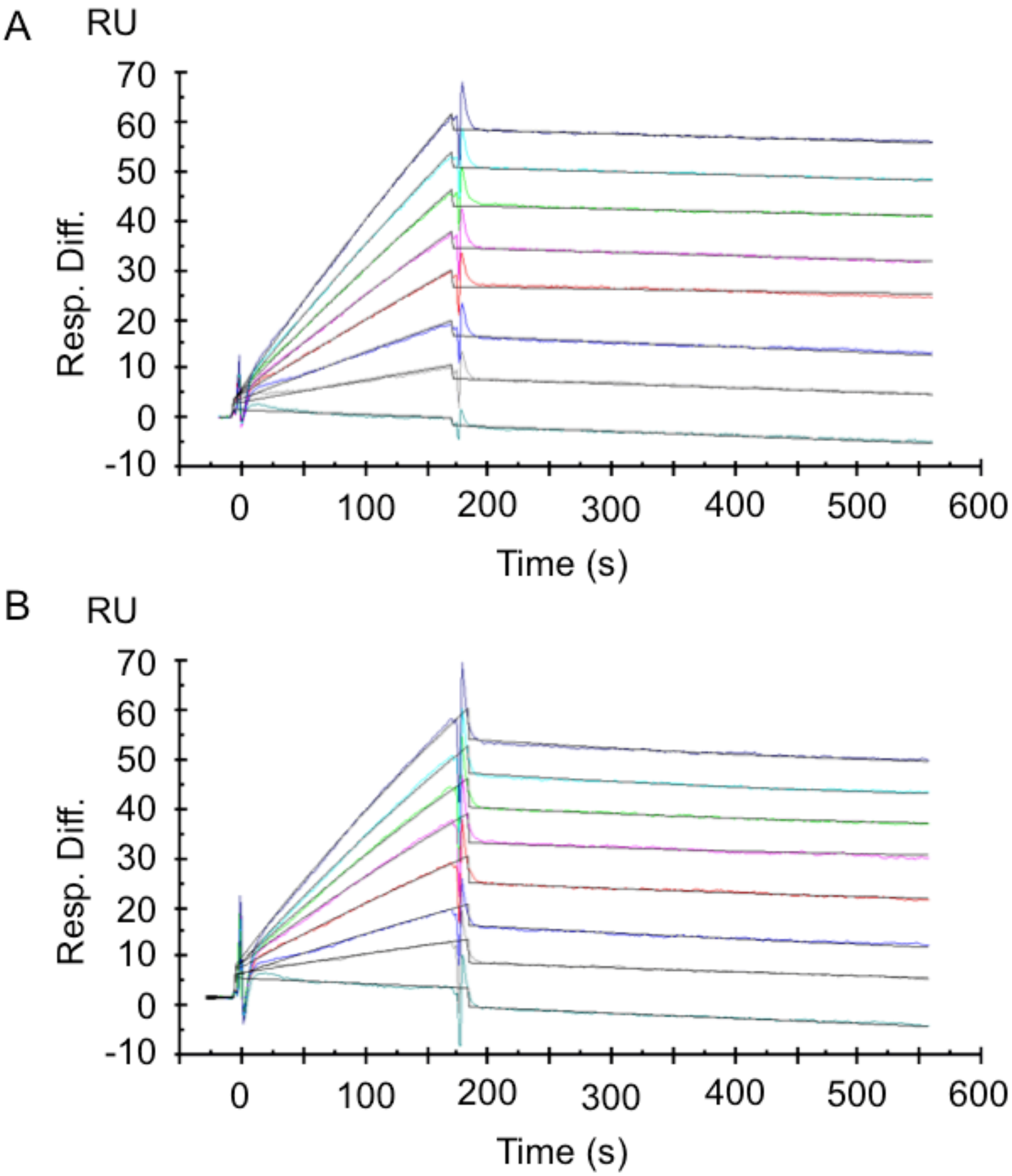

C

	kon (1/Ms)	koff (1/s)	KD (M)
**BSA-W614A-3S**	4.10e5	1.09e-6	2,65e-12
**BSA-3S**	8.06e5	8.11e-4	1.01e-9

Supplemental Figure 4: SPR sensorgrams of F8 mAb binding to BSA-W614S-3S and BSA-3S. The resonance signal in RU is given as the response difference (Resp. Diff.) between the reference cell (without immobilized ligand) and the sample cell. The F8 mAb was immobilized onto a CM5 sensor chip (as mentioned in Supplemental methods) and the analytes were injected at concentrations of 2.8, 2.4, 2, 1.6, 1.2, 0.8, 0.4 nM for BSA-W614A-3S (**A**) and 1.4, 1.2, 1, 0.8, 0.6, 0.4, 0.2 nM for BSA-3S (**B**). Binding rate constants and apparent affinities of BSA-3S and BSA-W614A-3S for the F8 mAb in SPR are shown in **C**.

Supplemental Table I: Molecular characterization of the anti-W614A-3S mAbs.

		B8 / G9	F8 / C9	G6
IGH	**V-gene and allele**	IGHV5-4*02<br>96% (276/288 nt)	IGHV3-6*01 or 02<br>93% (267/285 nt)	IGHV1-26*01<br>99% (268/271 nt)
	**J-Gene and allele**	IGHJ3*01<br>100% (48/48 nt)	IGHJ2*01<br>83% (40/48 nt)	IGHJ2*01<br>90% (43/48 nt)
	**D-Gene and allele**	IGHD1-1*01	IGHD2-2*01	IGHD2-12*01
	**CDR3 length**	11	5	10
	**CDR3 MW**	1,752.93	911.01	1,417.48
	**CDR3 sequence**	CTRDRDFSWFAYW	CYGYGYW	CARGNFGNDDYW
IGK	**V-gene and allele**	IGKV1-117*01<br>99% (291/294 nt)	IGKV1-117*01<br>96% (283/294 nt)	IGKV1-117*01<br>98% (289/294 nt)
	**J-Gene and allele**	IGKJ5*01<br>95% (36/38 nt)	IGKJ2*01<br>84% (32/38 nt)	IGKJ5*01<br>100% (36/36 nt)
	**CDR3 length**	9	9	9
	**CDR3 MW**	1,235.43	1,333.49	1,235.43
	**CDR3 sequence**	CFQGSHVPLTF	CFQGSHFPYTF	CFQGSHVPLTF

Supplemental Table II: Sequence of the peptides used in this study

3S-SCR: C W D A L N D W S P S K I A S N

3S: C P W N **W** S A S N K S L D D I W

W614A-3S: C P W N **A** S A S N K S L D D I W

Mim_F8-1: D L F Y **E C A G C** K

Mim_F8-2: **E C A G C** A V D S L

SUPPLEMENTAL MATERIAL AND METHODS:

Peptides Synthesis

The peptides described in the Supplemental Table II were synthesized by CovalAb (Villeurbanne, France) using solid-phase Fmoc chemistry with >80% purity levels. The biotinylated Mim_F8-1 peptide (DLFYECAGCK-Biot) was synthesized via Fmoc chemistry using the Liberty Blue™ automated microwave peptide synthesizer (CEM Corporation), a Biotin NovaTag (Merck Millipore), and a systematic double-coupling protocol. The peptide was cleaved from the resin and deprotected using standard TFA procedures with 1,2-ethanedithiol, water, and triisopropylsilane as scavengers. The peptide was purified by reverse-phase high-performance liquid chromatography (RP-HPLC) using a Phenomenex Luna® C18(2) semi-preparative column (5 *μ*m, 250 × 10 mm) and lyophilized. Pure peptide was dissolved with water/DMSO (2/1) and incubated at room temperature for 16h. The oxidized reaction mixture was purified directly by RP-HPLC and lyophilized. The homogeneity and identity of the synthetic peptide were assessed by MALDI-TOF mass spectrometry (Voyager DE-PRO Applied Biosystems) and analytical RP-HPLC (Grace Apollo C18 column, 5 µm, 250 x 4.6 mm).

Molecular characterization of mAbs

RNA was extracted from 10^6 hybridoma cells with Trizol reagent (Thermo Scientific, Ambion), and reverse transcription done using the Reverse Transcriptase Core kit (Eurogentec) according to manufacturer's instructions. The amplification of the IgG variable domains was performed as previously reported (1). DNA sequencing of purified

amplicons was performed on an ABI Prism 3700 automatic sequencer (Thermo Scientific, Applied Biosystems) using the BigDye Terminator v1.1 Cycle Sequencing Kit and analyzed with the Sequencher1v4.7 sequence analysis software (Gene Codes Corporation). Identification of V, D, J genes and alleles of Ig was performed with IMGT/V-QUEST program version: 3.3.5 (2, 3).

mAb chimerization

The IgG variable domains were amplified by PCR using the FastStart High Fidelity PCR System (Roche) from the cDNA of the F8 hybridoma using specific PAGE-purified oligonucleotides (Eurogentec). The VH and the VL domains were cloned into the pFUSEss-CHIg-hG1 and pFUSE2ss-CLIg-hk respectively (both from Invivogen) using the In-Fusion HD Cloning Kit (Takara Bio) according to the manufacturer instructions. After cloning, isolated colonies were screened by PCR and sequenced to check the integrity of the murine variable domains and the in-frame junction with the sequence of the human Ig constant domains.

CHO-K1 cells were co-transfected with the pFUSEss-***F8VH***-CHIg-hG1 and pFUSE2ss-***F8Vk***-CLIg-hk at a 2:3 ratio using JetPrime transfection reagent (Polyplus-Transfection) and selected with blasticidin (100 µg/mL) and zeocin (25 µg/mL) (both from Invivogene). Resistant cells were subcloned by limiting dilution in 96-well plates and supernatants were analyzed for chimeric antibody expression by indirect ELISA. One of the positive clones was culture expanded into DMEM supplemented with 2.5% of Ultra Low IgG Fetal Bovine Serum (Thermo Scientific, Gibco) and the chimeric antibody was purified from 100 mL supernatant using protein A agarose beads (Calbiochem).

Antibody binding assays and KD determination

Kinetics and affinity of the F8 antibody binding was analyzed by Surface Plasmon Resonance (SPR) on a Biacore 3000 instrument (GE Healthcare) using a CM5 sensor chip in order to obtain 1500 RU of covalently coupled protein essentially as described in (4). All experiments were performed in triplicate. The purified F8 mAb was immobilized at 30 µg/mL following by injection of 1M ethanolamine hydrochloride. Kinetic experiments were carried out in running buffer by passing various concentrations of the analyte over the ligand surface with a 3-min association phase and a 6-min dissociation phase. The sensor surface was regenerated between each cycle with 10 mM glycine hydrochloride (pH 2.5). The BSA-3S-scr was used as negative control and identical injections over blank surfaces were subtracted from all experiments. Kinetics were evaluated by using the BIAevaluation software, Version 4.1 (GE Healthcare). The data were processed by fitting the binding profiles to a 1:1 Langmuir interaction model. The quality of the fit was assessed by the statistical chi2 value provided by the software. The fitting of each dataset yielded rates for association (kon) and dissociation (koff), from which the equilibrium dissociation constant KD was calculated (KD = kon/koff). The kon, koff and KD from 3 experiments were used to calculate the mean values of these variables.

Biolayer interferometry (BLI) analyses were run on a BLItz instrument (ForteBio, Inc.). Streptavidin (SA) and anti-mouse IgG Fc Capture (AMC) biosensors were used to immobilize biotin-conjugated Mim_F8-1 peptide and mAbs, respectively, before to be dipped into solution of analytes diluted in kinetics buffer. Results were analyzed using

the BLItz Pro software (version 1.2.1.3), resulting in estimation of association and dissociation rates.

REFERENCES

1. Dorgham K, Dejou C, Piesse C, Gorochov G, Pene J, Yssel H. 2016. Identification of the Single Immunodominant Region of the Native Human CC Chemokine Receptor 6 Recognized by Mouse Monoclonal Antibodies. Plos One 11.
2. Brochet X, Lefranc MP, Giudicelli V. 2008. IMGT/V-QUEST: the highly customized and integrated system for IG and TR standardized V-J and V-D-J sequence analysis. Nucleic Acids Res 36:W503-8.
3. Giudicelli V, Brochet X, Lefranc MP. 2011. IMGT/V-QUEST: IMGT standardized analysis of the immunoglobulin (IG) and T cell receptor (TR) nucleotide sequences. Cold Spring Harb Protoc 2011:695-715.
4. Tudor D, Yu H, Maupetit J, Drillet AS, Bouceba T, Schwartz-Cornil I, Lopalco L, Tuffery P, Bomsel M. 2012. Isotype modulates epitope specificity, affinity, and antiviral activities of anti-HIV-1 human broadly neutralizing 2F5 antibody. Proc Natl Acad Sci U S A 109:12680-5.
